# Supplementary material for: The interactome of intact mitochondria by cross-linking mass spectrometry provides evidence for coexisting respiratory supercomplexes
Source: Mol Cell Proteomics. 2017 Dec 8;17(2):216–32. doi: 10.1074/mcp.RA117.000470 (PMC5795388; doi:10.1074/mcp.RA117.000470)
Supplement: Supplemental Data [file supp_RA117.000470_133922_0_supp_23978_fzffwf.zip › spectra_annotation/mito_DR_spectra_annotation/327-1-5-1-4-1.pdf]

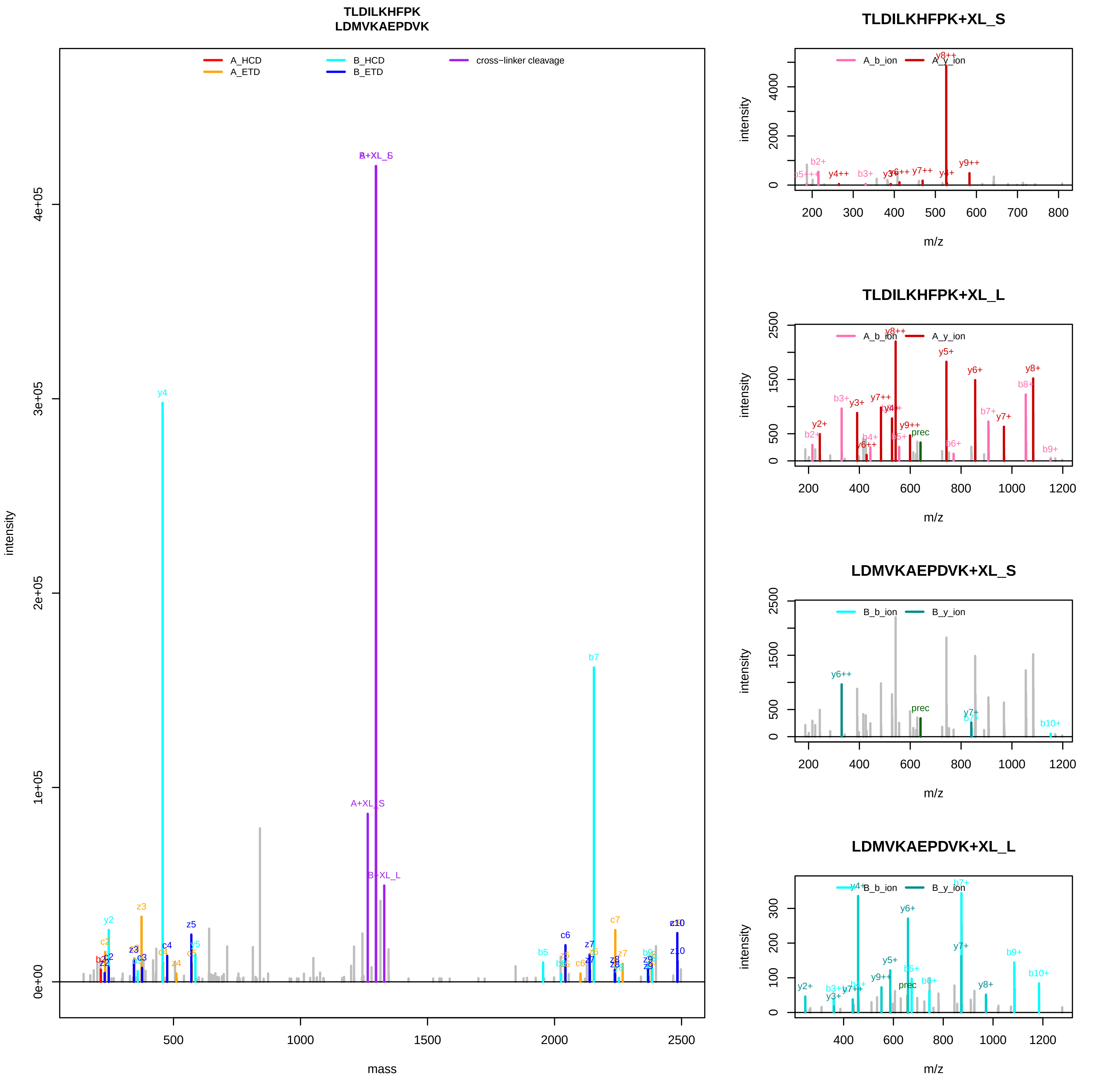

TLDILKHFPK
LDMVKAEPDVK
A_HCD
A_ETD
B_HCD
B_ETD
cross−linker cleavage
intensity
mass
0e+00
1e+05
2e+05
3e+05
4e+05
500
1000
1500
2000
2500
A+XL_S
B+XL_S
B+XL_L
y4
b7
y2
c2
z3
z2
c3
c4
z5
y5
c5
z4
b5
c6
z6
c7
z7
z8
z9
z10
c10
TLDILKHFPK+XL_S
A_b_ion
A_y_ion
m/z
y8++
b2+
y4++
b3+
y3+
y6++
y7++
y4+
y9++
TLDILKHFPK+XL_L
y5+
y6+
y8+
b8+
b7+
y7+
y2+
b4+
b5+
b6+
b9+
prec
LDMVKAEPDVK+XL_S
B_b_ion
B_y_ion
y6++
y7+
b7+
b10+
LDMVKAEPDVK+XL_L
y4+
y6+
y5+
y9++
b5+
b6+
b7+
y7+
b9+
b10+
y8+
y2+
b3+
y3+
y7++
